# Rethinking agrarian livelihoods affected by narcotic drug abuse on China’s Southeast Asian borders: a typological perspective

**DOI:** 10.3389/adar.2024.12693

**Published:** 2024-05-09

**Authors:** Xiaobo Hua

**Affiliations:** College of Humanities and Development Studies, China Agricultural University, Beijing, China

**Keywords:** livelihood, narcotics, borderland, typology, China

## Introduction

Narcotic drug abuse is one of the most serious social problems in the world, and China is highly vulnerable to its negative consequences [1–3]. From a supply chain perspective, narcotics from an illicit opium-producing area in the infamous “Golden Triangle” [4], pose one of the largest threats to the Chinese border and inland areas. There is a high frequency of cross-border drug trafficking and crime, especially along the Yunnan and Guangxi Provinces. As van Schendel and Abraham argued, the border is a place of extreme anxiety for the modern state due to a poor understanding of the notion of illegality [5].

The Chinese government has made great efforts to address and control these drug problems, but still faces major challenges in the fight against drug trafficking and crimes due to transnational illicit flows, especially in China’s Southeast Asian borderlands, where there is a long history of opium/heroin production, consumption, and trade [6–8].

In this context, much of the debate about narcotic drug problems in this border area is concerned with how to control supply [9], while we know much less about how narcotic drug problems affect or change the livelihoods of drug users and societies on the consumer side. In fact, many drug users in this region live in rural areas and work in agriculture [10]. There is therefore a gap in research concerning their livelihoods after drug abuse or cessation. Unlike other threats from non-traditional security issues, such as climate change or serious diseases, only certain or targeted villagers may suffer from drug abuse, and among these villagers, some of them may recover after receiving medical treatment. However, this does not mean that those who recover have completely stopped abusing drugs and it is still possible for them to relapse. In addition, drug users are not easy to locate and apprehend immediately because of their high mobility and ability to hide. Therefore, a better understanding of these issues will be useful when thinking about cross-border/transnational linkages, border governance, and sustainable rural development issues in the borderlands and other regions facing similar challenges.

## Reconsidering the characteristics of drug users

Unlike conventional approaches to understanding rural populations, such as those based on gender, age, or wealth, there are specific characteristics associated with drug users. Apart from those who are still abusing drugs, the majority of former drug users require medical treatment in order to recover. In terms of medical treatment and relapse, drug users normally lose the physical feeling of addiction once they are forced to stay in a rehabilitation center and can fully recover after receiving 1 month of continuous and professional treatment. However, the case of compulsory punishment is different. These recovered drug addicts are very likely to relapse when they are released. There are a few possible reasons for this: 1) Physical and mental dependence on drugs results from physiological damage after drug addiction. For example, being addicted to heroin only once can result in a strong mental dependence on drugs. 2) They are easily affected by items related to drug abuse, such as a syringe and aluminum foil, which may encourage them to abuse drugs again. 3) Widespread discrimination from their families and society can trigger a relapse. 4) They are easily disappointed in themselves if it is relatively difficult for them to thrive when re-entering society. In this complicated context, it is imperative to understand the agrarian livelihoods affected by narcotic drugs rather than comparing them to those who have never abused drugs.

## Typologies of agrarian livelihoods affected by narcotic drugs

**FIGURE 1:**
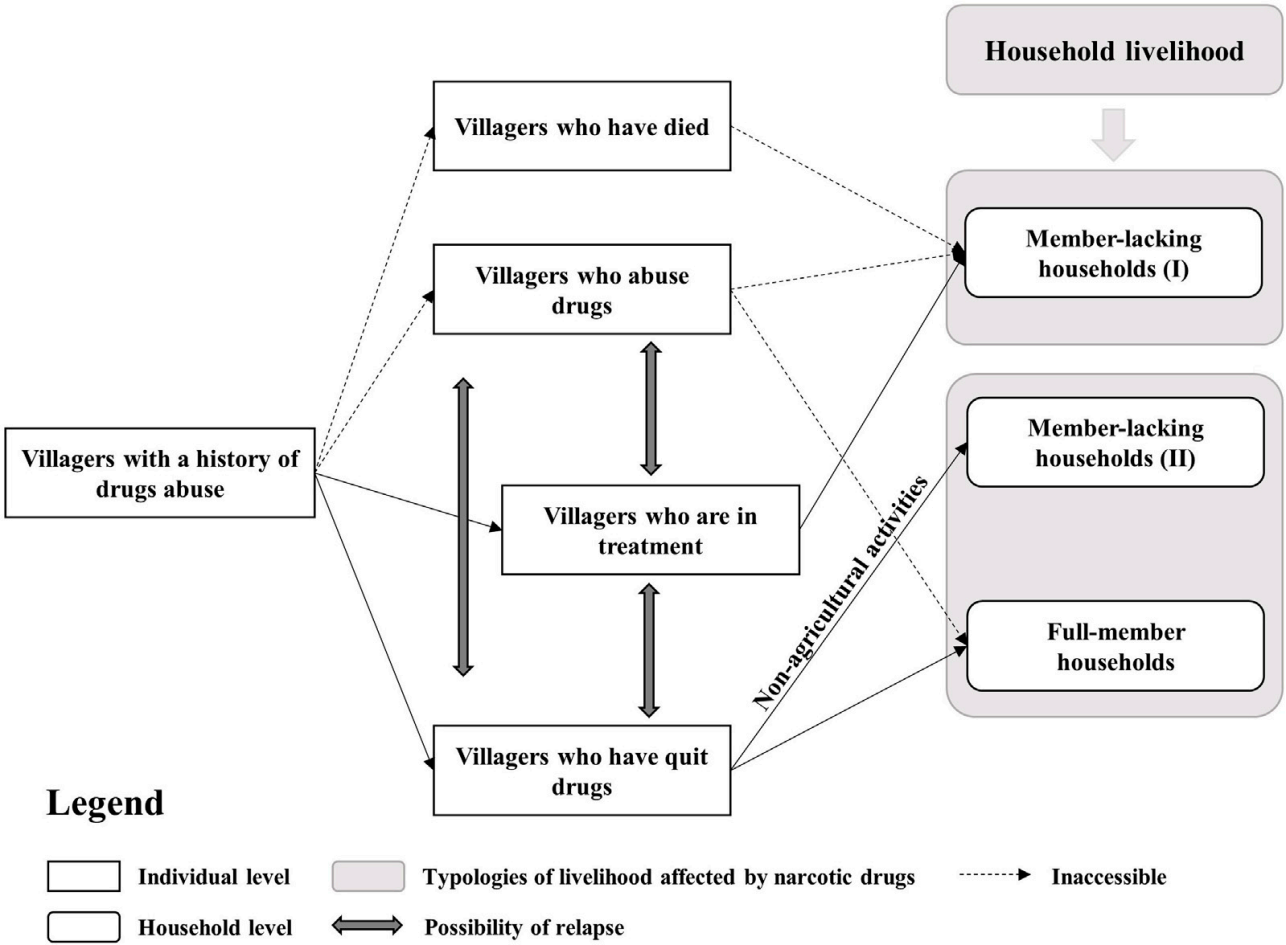
A typology linking villagers having experienced drug abuse and agrarian livelihoods.

For a better understanding of how narcotic drugs have affected the local villagers and their households, this paper proposes four typologies of villagers with drug abuse experience based on the characteristics of drug abusers (Figure 1). The first group included villagers who passed away due to drug addiction. The second group was made up of villagers who were currently abusing drugs. However, these two groups were very difficult to access and interview because of practical and ethical considerations. The third group included villagers who were under treatment in the drug addiction treatment center. These were villagers who had been using drugs for a long time and had been sent to the drug treatment center. The fourth and last group consisted of villagers who had stopped using narcotic drugs after treatment. Villagers belonging to the last group had returned to lead normal lives. Nevertheless, there is a possibility of relapse among the villagers who are under treatment or have completed treatment because of their strong dependence on narcotic drugs both mentally and physically. In general, there was a high possibility of recurrence and uncertainty among the last three groups.

The villagers belonging to the above groups generally formed two types of households (Figure 1). The first type included member-lacking households (MHHs), which means that the previous and normal household structure may have been affected and some family members may have left because of drug addiction. In an MHH, some members of a household may have passed away, some may still be abusing drugs, some may be in treatment, or some may have left treatment to seek non-agricultural opportunities. The absent family member resulted in a potential labor shortage at home and may not have directly contributed to the household’s operation. Therefore, MHHs were further divided into two sub-types: MHHs-I and MHHs-II. MHHs-I represented households with members who experienced drug abuse and could not contribute to the household, while MHHs-II referred to households with members who were drug abusers but worked as agricultural or non-agricultural laborers. The second type of household was the opposite of the first: full-member households (FHHs). This may include members who were stillabusing drugs and living with their families or members who had quit drug addiction for several years and mainly made a living through agricultural activities or local work. Many studies have shown that labor capacity, education, and health levels have a positive impact on off-farm employment or engagement in non-agricultural work. However, it remains unclear whether these factors contribute to the ability of former drug users to participate in non-agricultural work.

## Discussion

Drug abuse in China’s Southeast Asian borderlands is not merely a drug issue, but an urgent social and cultural agenda. It has significantly affected rural society on the Chinese side as a receiving frontier for illicit flows of narcotic drugs. From a typological perspective, this opinion article focuses on the characteristics of villagers and their respective households that are heterogeneously affected by narcotic drugs. The proposed typology is relevant to the existing literature on rural livelihoods, in addition to policies for governance, poverty alleviation, and sustainable rural development under the influence of narcotic drugs in China’s Southeast Asian borderlands and other regions facing similar challenges. It also highlights some practical considerations. First, as mentioned in the typology section, it is difficult to study the impact of narcotic drug problems on local livelihoods and we must be careful of the interviewees’ feelings when asking about drug addiction in the household. This is because such questions could easily make the interviewees feel ashamed, embarrassed, or sad. Second, this proposed typology mainly focuses on the characteristics of the villagers with prior experience of drug abuse and their households, rather than comparing the households with and without members who have previously or currently dealt with drug abuse. Such comparison would be much more complicated. For example, some villagers may be renting out their farmland not only because their labor capacity has been negatively influenced by drug abuse or disease, but also because of the costs and benefits of farming activities or as land tenants. These factors may overlap. Such situations should be further clarified and discussed in future research.
